# Genetics and genomic medicine in Cuba

**DOI:** 10.1002/mgg3.299

**Published:** 2017-05-21

**Authors:** Hilda Roblejo Balbuena, Beatriz Marcheco Teruel

**Affiliations:** ^1^ National Centre of Medical GeneticsMedical University of HavanaHavanaCuba

## Abstract

Genetics and genomic medicine in Cuba.

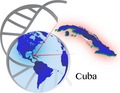

## Introduction

The Republic of Cuba is an archipelago formed by more than 1600 islands, islets and keys, with the Island of Cuba being the greater one. Its geographic location includes 19°49′ and 23°16′N and 74°08′ and 84°57′W, from the Greenwich meridian; that places it north of the Caribbean Sea and south of the Tropic of Cancer, at the entrance to the Gulf of Mexico (National Office of Statistic and Information, ONEI, www.one.cu/cuba2010.htm).

**Figure 1:**
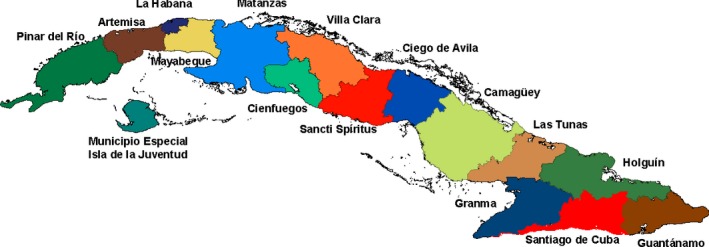
Political map of Cuba.

The surface area of the Cuban archipelago is 109,884.01 km^2^ (Fig. 1). Its population is settled in the islands of Cuba and the Youth Island, the rest of the archipelago is almost depopulated with the exception of tourist centers in some keys. Its capital is Havana. As of 2011, a new political‐administrative division was established, with which Cuba was organized into 15 provinces and 168 municipalities, including the special Youth Island municipality (Isla de la Juventud). The country has a population of 11,239,004, of which more than 75% live in urban areas (Statistical Yearbook 2015. ONEI. http://www.one.cu).

Cuba is recognized internationally for its high educational and cultural levels, as well as for the qualified provision of its health services. The infant mortality rate was 4.3 per thousand live births at the end of 2016, which places the island in the vanguard of America and among the first 20 nations of the world. Life expectancy at birth is 78.45 years for both sexes (76.5 for men and 80.45 for women) (Statistical Yearbook 2015. ONEI. http://www.one.cu).

## Health Services in Cuba

The National Health System is one of the greatest achievements of the Cuban social model, that includes among its guiding principles the state and social nature of medicine, accessibility and gratuity of services, prophylactic guidance, adequate application of advances Science and technology, community participation, intersectorality, and international collaboration (Álvarez Pérez et al. 2007). Medical services are provided on three levels of care depending on the complexity of the specific institution: primary, secondary, and tertiary care.

## The National Program for the Diagnosis, Management and Prevention of Genetic Diseases and Birth Defects in Cuba

The services of medical genetics are integrated under the National Program for Diagnosis, Management and Prevention of genetic diseases and birth defects. This program started in the 1980s. The services are implemented through a community genetics network that currently has 168 municipal genetics services, 15 provincial services and the National Center of Medical Genetics (NCMG) in Havana. The national program is coordinated by the NCMG; the national reference center is also responsible for undergraduate and postgraduate training, research, and introduction of new technologies in this field (Marcheco‐Teruel 2009a). The NCMG is a World Health Organization Collaborative Center for the Development of Genetic Approaches to Health Promotion.

Medical genetics in Cuba includes programs as part of prenatal, neonatal and postnatal care (Table 1).

**Table 1:** Healthcare programs available in the medical genetics services for the primary, secondary and tertiary healthcare levels in Cuba

Life stage	Healthcare program
Prenatal	Prevention of sickle‐cell anemia by the detection of carriers and prenatal diagnosis.
	Detection of congenital defects by the quantification of alpha‐fetoprotein in maternal serum.
	Detection of congenital defects by ultrasound techniques in the 1st, 2nd and 3rd trimesters of pregnancy.
	Prenatal diagnosis of chromosomal anomalies for pregnancies with increased genetic risk.
Neonatal	Neonatal screening of phenylketonuria, galactosemia, biotinidase deficiency, congenital hypothyroidism, congenital adrenal hyperplasia.
	Evaluation of the newborn at birth and in the first three months of life.
Postnatal	Clinical diagnosis of genetic diseases.
	Genetic counseling in common diseases.

*Source:* Marcheco‐Teruel (2009b). The national program for the diagnosis, managing and prevention of genetic diseases and birth defects in Cuba: 1981–2009. Rev Cubana Genet Comunit. 3(2–3):167–84.

The program of prevention of sickle‐cell anemia (Hb SS/HBSC) by detecting carriers and prenatal diagnosis began in 1983. It is based on HbSS and HbSC screening, providing the study of hemoglobin electrophoresis to all pregnant women in the first trimester of pregnancy. The program includes procedures for the identification of high‐ risk couples, the provision of genetic counseling, and the availability of prenatal diagnosis for molecular studies of the gene. Couples at high risk of having children affected by this disease, can obtain information, have access to services and the necessary resources to adopt reproductive behavior appropriate to their interests (Martín‐Ruíz et al. 2008).

According to data from the Institute of Hematology and Immunology of the Ministry of Public Health, approximately 5000 people throughout the country suffer from sickle cell anemia. The frequency of sickle cell trait (HbAS) in the Cuban population was reported at 3% and the trait for Hemoglobin C (HbAC) at 0.7% (Domínguez‐Mena et al. 2005 and Martín‐Ruíz et al. 2008). Sickle cell anemia is considered a health problem in Cuba. The most Eastern region of the country has a higher proportion of genes from African ancestry (Marcheco‐Teruel et al. 2014), so that in this area the proportion of high‐risk couples is higher than the national average.

Another subprogram implemented in Cuba since 1982 is the screening for congenital defects based on the determination of maternal serum alpha fetoprotein (AFP), which includes the use of ultrasound for the study of cases with elevated values. The determination of AFP is made during gestational age 15–19 weeks (Llanusa‐Ruiz et al. 1998). As an average, the existence of a congenital defect has been detected in one out of every 500 pregnancies with this study in the whole country. The program coverage has exceeded 95% of the country's pregnant women since 1995, (Marcheco‐Teruel 2009b). Between 2 and 3% of the elevated AFP correspond to structural closure defects, including those of neural tubes, congenital anterior abdominal walls and others such as bilateral renal agenesis (Llamos‐Paneque et al. 2007).

Also in the early 1980s, the practice of ultrasound for prenatal diagnosis of congenital malformations was introduced in Cuba. This program has been decentralized to the primary level, increasing access to it since 2004. Currently, all pregnant women have planned an ultrasound in the first trimester between 11 and 13.6 weeks of pregnancy, something highly useful for the diagnosis of the possible presence of aneuploidies and other malformations in the fetus. In the second trimester of pregnancy, all pregnant women have access to another ultrasound that is performed between 20–22 weeks. In this ultrasound study, the greatest number of diagnoses corresponds to renal defects, followed by congenital heart disease and other malformations of the central nervous system (Marcheco‐Teruel 2010). Since 2013, a third genetic ultrasound is universally available between 28–32 weeks of gestation.

As part of prenatal care, antenatal cytogenetic testing is also planned for pregnant women at risk, including those of an advanced maternal age. It aims to provide the opportunity to pregnant women with higher risk of having offspring affected by chromosomal defects, to know in the prenatal stage if fetus is affected, so that reproductive decisions can be taken by the couple according to their interests. Higher risk pregnancies include those where the maternal age is 37 or older, previous offspring with Down syndrome or another chromosomopathy, pregnant women with suspected ultrasonographic markers associated with chromosomopathies, as well as pregnant women with a family history of chromosomal rearrangements (Falcón‐Fonte et al. 2015).

A recent study undertaken by Méndez‐Rosado et al. (2016) collected data of a total of 75,095 prenatal cytogenetic diagnoses in the period 1984–2012, of which 68,206 corresponded to amniocyte culture, 6311 to chorionic villus sampling, 407 to cordocentesis and 171 cases performed by fluorescence in situ hybridization in interphase cells. The aneuploidies of chromosomes 21, 18, 13, X and Y constituted 76.8% of all chromosomal abnormalities detected. Down Syndrome represented 47.2% of all chromosomal aberrations. In 2002, the prevalence of Down syndrome in Cuba was 8.4 per 10,000 live births and in 2012 it fell to 7.0 per 10,000 live births, one of the lowest reported in international scientific literature (2016).

The national neonatal screening program is coordinated by the National Center of Medical Genetics. It is characterized by a design that ranges from collecting the sample of the newborn in the primary level of health care, to the diagnosis and follow‐up of patients confirmed at the secondary and tertiary levels.

This screening program started in 1983 with the neonatal detection of phenylketonuria using dried blood samples on filter paper to measure phenylalanine concentration by the Guthrie‐Susi method. The program was expanded to the rest of Cuba in 1986. In 2000, a new technology was introduced in the research using the UMTEST‐PKU diagnostic kit of Cuban production, through the Ultra Micro Analytical System (SUMA) (Martínez‐Rey et al. 2008). In 1986, the newborn screening for congenital hypothyroidism was started from umbilical cord blood at the time of birth. This program is coordinated by the National Institute of Endocrinology. In 2006, three new diseases were added to the newborn screening through the Ultra Micro Analytical System: biotinidase deficiency, galactosemia and congenital adrenal hyperplasia (Marcheco‐Teruel 2009b) (Table 2).

**Table 2:** Prevalence of disorders among Cuban newborns

Disorder	No. of confirmed cases	Incidence	Period	Source
Congenital adrenal hyperplasia	51	1:21,136	2005–first semester 2014	Reyes (2016)
Phenylketonuria	9	1:32,000	2014–2015	National Records available at the National Center of Medical Genetics
Biotinidase deficiency	10	1:110,032	2006–2014	Moreno‐Arango et al. (2016)

In addition to prenatal and neonatal care, clinical genetics services have been incorporated in pediatric or adult hospitals and in tertiary care centers, such as the National Institutes of Ophthalmology, Neurology, Cardiology, Oncology, Endocrinology, and Gastroenterology. These services attend to patients and families with genetic diseases of monogenic, chromosomal or multifactorial etiology.

The five labs (Molecular Biology, Biochemical Genetics, Immunology, Cytogenetics and Oxidative Stress) of the National Center of Medical Genetics, the leading institution of the genetics program, use the most advanced technology available in the country, to guarantee the diagnosis of more than 150 genetic diseases (http://instituciones.sld.cu/cngm/).

## Training of Human Resources

### Undergraduate teaching of medical genetics in the career of medicine

The teaching of Genetics in undergraduate courses began in the early 1970′s academic years, from 1971 to 1972. Actually, as part of the continuing improvement of the medical career program, the impact of the results of the Human Genome Project on medical genetics and on primary health care, the Genetics Medical course is taught in the fourth semester of the medical degree and the program includes 54 teaching hours (Lantigua‐Cruz 2009).

### Specialization program in clinical genetics

In 1977, Clinical Genetics was recognized as a medical specialty in Cuba according to Ministerial Resolution No.33/1977 (Lantigua‐Cruz 2009). Specialization is offered to medical doctors and takes four years. It includes a basic cycle, a clinical cycle with rotations comprising the specialties of pediatric neurology, neonatology, ophthalmology, dermatology, endocrinology, cardiology, orthopedics, prenatal care as well as laboratory training in molecular biology, biochemical genetics, and cytogenetics.

### Master's programs in genetic counseling and medical genetics

From 2002, there was a significant leap in the development of community genetics in the country, with the training of genetic counselors through a master's degree in genetic counseling. This made it possible to increase the coverage of care services in medical genetics, primary care along with the creation of services for the development of Community Genetics in all municipalities of the country (González‐Lucas and Lantigua‐Cruz 2007). The Master's Program in Genetic Counseling includes a performance profile in medical genetics services to the community.

The Master's Program in Medical Genetics is intended to provide physicians and practitioners involved in the care of genetic diseases and birth defects with new updates on the approaches, concepts and knowledge that have emerged in light of the Human Genome Project, with great impact on the medicine. Both masters degrees are imparted at the National Center of Medical Genetics.

## Research in the Field of Medical Genetics

One of the most important lines of research in the field of Medical Genetics has been the clinical‐genetic approach to characterize the genetic etiology of intellectual disability. The prevalence rate of this disability in Cuba is 1.25 per 100 inhabitants (Cobas‐Ruíz et al. 2011).

The development of the bases for genomic medicine in order to improve the standard of individual health care involves the study of the population's genetic origin and structure. It is necessary to take into account the genomic admixture and genetic stratification of the population when designing association studies for common diseases. In the Cuban population, the average European, African, and Native American contributions as estimated from Autosomal Ancestry Informative Markers (AIMs) were 72%, 20% and 8%, respectively (Marcheco‐Teruel et al. 2014).

Since 1985, the Cuban Registry of Congenital Malformations (RECUMAC) has been used as a research program for congenital defects that allows establishing a system of clinical and epidemiological surveillance of the same in the hospital births. This record includes all live births or stillbirths of babies weighing 500 grams or more that have one or more birth defects. This is a specialized and highly complex data file (Pérez‐Mateo and Fuentes‐Smith 2007). The information in this registry continues to grow, covering up to 96% of the total births in the country. In Cuba there are between 110 thousand and 120 thousand births per year.

The molecular study of monogenic diseases has been another line of research. In the case of cystic fibrosis seven common CF mutations (p.F508del, p.G542X, p.R1162X, p.N1303K, p.R334W, p.R553X and c.3120+1G>A) were investigated, taking into account the ethnic origin of the Cuban population which is mainly influenced by Spanish and sub‐Sahara African contributions. All but p.N1303K were detected in the patients, the p.F508del being the most prevalent (37.9%). Overall, six mutations showed frequencies above 1% accounting for 55.5% of the Cuban CF alleles (Collazo et al. 2009).

Other diseases have been molecularly characterized by direct and indirect methods such as fragile X syndrome (Lardoeyt Ferrer and Lantigua Cruz 2004; and Lantigua‐Cruz, et al. 2007) , Wilson disease (Clark Feoktistova et al. 2011), hemophilia A and B (Piloto et al. 2010), various types of hereditary nonsyndromic deafness (Morales‐ Peralta et al. 2007 and Álvarez et al. 2009), hemochromatosis (Cervera García et al. 2013), and alpha*‐*1 antitrypsin deficiency (González et al. 2012). These investigations open up a spectrum of possibilities, offering excellent new approaches to research and clinical genetics services, in function of the prevention of these genetic diseases.

Cuba is one of the countries with high rates of prevalence and incidence of hereditary ataxias, which is a health problem that encouraged the foundation of the Center for Research and Rehabilitation of Hereditary Ataxias in Holguín province (CIRAH). Specifically, spinocerebellar ataxia type 2 (SCA2) reaches the highest prevalence rate in the world. The first epidemiological observations were made between the decades of the 60s and 70s of the last century, and since then a predominance has been identified in the eastern region of the country. The investigations carried out in Cuba and specifically in the CIRAH have been of great importance for the study of the genetic and molecular characteristics of the SCA2 (Velázquez‐Pérez et al. 2011).

In the field of genetic epidemiology, research has been designed using the Cuban registry of twins, which have allowed the estimation of heritability in multifactorial diseases such as asthma, hypertension, coronary heart disease, schizophrenia, major depression, bipolar disorder, alcohol abuse or dependence. The database contains extensive information from 55,400 twin pairs enrolled in the period 2004–2006 (Marcheco‐Teruel et al. 2013).

## Ethical and Legal Framework

Ministerial Resolution 219 of 2007, published in the Official Gazette of the Republic of Cuba, establishes the Ethical Norms for the protection of genetic information of Cuban citizens who participate in investigations or who are diagnosed with a condition in which access to data relating to the individual and their families, as well as biological material from which DNA can be obtained (http://www.sld.cu/galerias/pdf/sitios/genetica/resolucion_219.pdf). (Rojas‐Betancourt et al. 2013).

## Final Remarks

The main strength of Genetics in Cuba, a low income country whose health expenditures consume a significant proportion of the annual budget, is the community approach, which has allowed strategies to be established for the detection and prevention of genetic risk. This has favored a progressive decrease in infant mortality due to birth defects, at values below 1 per 1000 live births from 2008 to date.

On the other hand, research in this field is aimed at the progressive introduction of applications in the clinical practice of genomics and related technologies, always complying with the principle of introducing their results for the benefit of the Cuban population.

Cuba has highly trained human resources in medical genetics, so that the generalization of genomic technologies and, fundamentally, the reduction in production costs, will allow, within a medium period of time, quicker transfer of the actions of personalized medicine to clinical practice.
